# Perceived racism or racial discrimination and the risk of adverse obstetric outcomes: a systematic review

**DOI:** 10.1590/1516-3180.2021.0505.R1.07042022

**Published:** 2022-08-29

**Authors:** Glaucia Miranda Varella Pereira, Veronica Maria Pimentel, Fernanda Garanhani Surita, Amanda Dantas Silva, Luiz Gustavo Oliveira Brito

**Affiliations:** I PT, MSc. Doctoral Candidate, Department of Obstetrics and Gynecology, School of Medical Sciences, Universidade Estadual de Campinas (UNICAMP), Campinas (SP), Brazil.; II MD, MSc. Attending Physician, Department of Obstetrics and Gynecology, Saint Francis Hospital and Medical Center-Trinity Health of New England, Hartford, Connecticut, United States; Assistant Professor, Frank H. Netter M.D. School of Medicine, Quinnipiac University, North Haven, Connecticut, United States; and Assistant Professor, School of Medicine, University of Connecticut (UConn), Farmington, Connecticut, United States.; Quinnipiac University, School of Medicine, North Haven, Connecticut, United States; University of Connecticut, School of Medicine, Farmington, Connecticut, United States; III MD, PhD. Associate Professor, Department of Obstetrics and Gynecology, School of Medical Sciences, Universidade Estadual de Campinas (UNICAMP), Campinas (SP), Brazil.; IV MD. Attending Physician, Department of Obstetrics and Gynecology, School of Medical Sciences, Universidade Estadual de Campinas (UNICAMP), Campinas (SP), Brazil.; V MD, PhD. Associate Professor, Department of Obstetrics and Gynecology, School of Medical Sciences, Universidade Estadual de Campinas (UNICAMP), Campinas (SP), Brazil.

**Keywords:** Pregnancy, Racism, Systematic review [publication type], Preterm birth, Racial discrimination, Racial prejudice

## Abstract

**BACKGROUND:**

Racial disparities are differences among distinct subgroups of the human species; biologically, there are no scientifically proven reasons for them to exist.

**OBJECTIVE:**

To assess the impact of racism or racial discrimination on obstetric outcomes.

**DESIGN AND SETTING:**

Systematic review conducted at a tertiary/academic hospital.

**METHODS:**

The Cochrane Library, SCOPUS/EMBASE, PubMed, Web of Science and ClinicalTrials.gov databases were searched from inception to June 2020. Studies presenting any type of racial discrimination, or any manifestation of racism that was perceived by women of any age in an obstetric scenario were included. Studies that only assessed racial disparities without including direct racism were excluded. The secondary outcomes evaluated included quality of antenatal care, intra and postpartum care, preterm birth and birthweight. The Risk of Bias In Non-randomized Studies - of Interventions (ROBINS-I) scale was used to assess the quality of evidence from non-randomized studies.

**RESULTS:**

A total of 508 records were retrieved and 29 were selected for qualitative synthesis. No meta-analysis could be performed due to the high heterogeneity across studies. Perceived racism was associated as a risk factor in 7/10 studies focusing on pregnancy and postpartum maternal outcomes, five studies on preterm birth, one study on small for gestational age and two studies on low birthweight. Overall, among the 29 studies, the risk of bias was classified as moderate.

**CONCLUSIONS:**

Perceived racism presented an association with poor obstetric outcomes. Anti-racist measures are needed in order to address the problems that are causing patients to perceive or experience racism.

**SYSTEMATIC REVIEW REGISTRATION:**

PROSPERO database, CRD42020194382

## INTRODUCTION

Evidence that racial and ethnic disparities are present in healthcare matters and that structural racism is involved as a key determinant of populations’ health is growing.^1^ Studies within obstetrics have shown that racial disparities influence maternal morbidity and mortality, and that non-Hispanic black women are at highest risk of these outcomes in addition to being at highest risk of entering antenatal care late and being insufficient users of healthcare assistance.^2^ In a recent systematic review, empirical studies provided evidence to show that race and ethnicity have a role in pregnancy-related mortality and severe maternal morbidity risk.^3^ However, the number of studies on racial disparities surpasses those on racism itself.

Racial disparities are differences among distinct subgroups of the human species. However, biologically, there are no scientifically proven reasons for them to exist. Nonetheless, race has social significance because it may be used within a system of domination and oppression within which one racial group receives benefits and privileges from systematic subjugation of other racial groups.^4^ Thus, racial disparities are the tip of the iceberg, as the effect is seen in relation to several disorders throughout medicine. In obstetrics, the effect of racism leads to racial disparities that involve not only the woman but also the newborn or the whole family.

Racism is defined as “an organized system, rooted in an ideology of inferiority that categorizes, classifies and allocates social resources to groups of the human population in different ways”.^5^ In addition to being considered to be a determinant of health, due to its dynamic nature that endures and adapts over time, thereby influencing policies and practices that affect health, racism reflects norms and practices that are perceived as common, constant and chronic.^6–8^ Therefore, it is important to study the effect of racism at every step of the way, in order to analyze outcomes that can lead to solutions.

## OBJECTIVE

We aimed to assess the impact of racism or racial discrimination within obstetric outcomes, considering that in obstetrics, the effect of racism may lead to racial disparities that involve both the woman and the child.

## METHODS

This systematic review was conducted in accordance with the PRISMA guidelines^9,10^ (Preferred Reporting Items for Systematic Reviews and Meta-Analyses). The protocol for this review was registered in the PROSPERO database (under the number CRD42020194382).^11^ The Cochrane, EMBASE/SCOPUS, PubMed, Web of Science and ClinicalTrials.gov databases were searched electronically on the same day (July 1, 2020) using Medical Subject Headings (MeSH) terms and entry terms, along with keywords and word variants, for the terms obstetrics and racism (https://www.crd.york.ac.uk/prospero/display_record.php?ID=CRD42020194382). There were no language or time-span restrictions.

### Study selection

This review included observational studies that reported any type of racial discrimination, or any racism manifestation perceived by women of any age in an obstetric scenario. We considered studies that measured manifestations of racial discrimination or racism using questionnaires, indexes or scales in association with obstetric outcomes. Studies within obstetrics or studies that considered racial disparities or racial inequalities within obstetrics that did not measure manifestations of racial discrimination or racism were excluded. We also excluded qualitative studies that did not present any quantitative data, in accordance with the inclusion criteria.

The primary outcome was the presence of perceived racism or racial discrimination, reported as a categorical answer (yes/no), or as the sum score from an instrument measuring racial discrimination or racism.

### Measurements

The following scales and indexes were investigated: Experience of Discrimination Scale,^12^ Daily Life Experiences of Racism and Bother Score,^13^ Racism and Life Experience Scale,^14^ Racial Segregation Index,^15^ Major Discrimination Scale,^16^ Index of Concentration at the Extremes,^17^ Perception of Discrimination During Childbirth,^18^ Gendered Racial Microaggressions Scale,^19^ Measure of Indigenous Racism Experience,^20^ Racism-Related Scale,^21^ Chronic Worry,^12^ Williams Scale of Everyday Discrimination^12^ and Perceived Racism Scale.^22^

The Experience of Discrimination Scale is a validated and reliable nine-item questionnaire that has been used in eleven studies. It is based on a previous seven-item instrument developed by Krieger et al. in 1990.^23,24^ This multi-item self-report instrument measuring experiences of racial discrimination presents nine-item questions about discrimination in several domains, including at school and work, and investigates the frequency of discrimination.^12^

The Experience of Discrimination questionnaire was validated in the American population through confirmatory factor analysis and the results showed adequate model-fit indices.^12^

We used a spreadsheet for data extraction that had previously been pilot-tested. It exhibited the following variables: author/year, subject, variables, the time when the interview took place, sample size and main results (with descriptive data or crude/adjusted analysis if the variables were estimating the effect of an association between racism/racial discrimination and a dependent variable).

### Data extraction

Two researchers (GMVP and LGOB) independently evaluated the titles and abstracts of screened articles. A full-text evaluation was performed when the abstracts did not provide sufficient methodological information. The two researchers also independently analyzed full-text articles to determine study eligibility and to extract data. A third reviewer (FGS) helped in cases of any inconsistencies in the data.

### Assessment of risk of bias

Study quality was assessed by two investigators independently using the Risk of Bias In Non-randomized Studies of Interventions (ROBINS-I) tool. The studies were judged in terms of bias as “low risk”, “moderate risk”, “serious risk”, “critical risk” and “no information”, for the following domains: confounding, selection of participants, classification, deviations from intended interventions, missing data, measurement of outcomes, reported result and overall bias.^25^

### Data synthesis

Interventions and outcomes were presented differently among the studies selected, which precluded meta-analysis (due to heterogeneity). The present analysis was therefore restricted to a systematic review. We divided the results according to maternal outcomes (maternal smoking, antenatal entry, antenatal stress, delayed antenatal care, maternal blood pressure, antenatal sleep quality, trust in providers, etc.) and neonatal outcomes (preterm birth, small for gestational age and low birthweight).

## RESULTS

**Figure 1:**
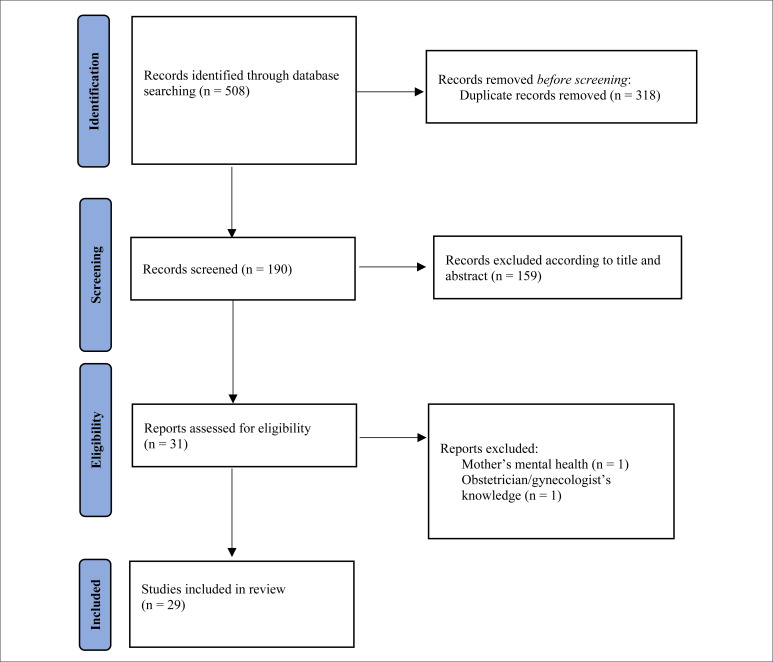
Flowchart of different steps of the systematic review.

The search strategy identified 508 articles; of these, two studies were excluded because they did not meet the inclusion criteria and 29 studies were included for final qualitative synthesis and are displayed in Figure 1. These comprised 16 cross-sectional studies, 11 cohort studies and two case-control studies. No randomized clinical trials were found regarding this subject. The number of participants per study ranged among the studies from 39 to 8,962 women.

The maternal outcomes (Table 1) included racial discrimination in pregnancy and childbirth. Four studies included antenatal care that involved racial discrimination with regard to smoking,^26^ perceived discrimination through delayed antenatal care,^27^ experience of racial discrimination in antenatal entry^28^ and racial discrimination regarding perceived antenatal stress/depression.^29^ Eleven studies on pregnancy assessed general perceived racism,^30–34^ racial discrimination in relation to Epstein-Barr virus reactivation,^35^ racism in relation to blood pressure changes,^36^ racism in relation to trust in providers,^37^ racial segregation with regard to smoking,^38^ perceived discrimination in maternity care^39^ and racial discrimination in relation to biological measurements.^40^ One study included racial discrimination with regard to perinatal sleep quality.^41^ Lastly, perceived discrimination during childbirth was reported in one study.^18^

**Table 1:** Racism or racial discrimination within studies comprising antenatal care, childbirth and postpartum period

Author, year/study design	Subject	Measurement	Time of interview	Sample size	Main results (descriptive or after crude/adjusted analysis)
Nguyen et al.,^26^ 2012/CS	Racial discrimination in relation to PN smoking	EODQ	Baseline interview at mean of 26.9 weeks of gestation	n = 677 -n = 265 black -n = 412 Hispanic	EODQ (whole sample) - moderate: (adjusted OR 1.00) - none: (adjusted OR 1.67; CI 0.86-3.21) - high: (adjusted OR 2.64; CI 1.25-5.60) EODQ (stratified) Hispanic women - moderate: (adjusted OR 1.00) - none: (adjusted OR 2.45; CI 0.90-6.70) - high: (adjusted OR 2.08; CI 0.60-7.14) Black women - moderate: (adjusted OR 1.00) - none: (adjusted OR 1.05; CI 0.42-2.62) – high: (adjusted OR 3.36; CI 1.23-9.19)
Slaughter-Acey et al.,^28^ 2013/CH	Racism in relation to PN care	DRI: RaLES	At 22 to 28 weeks of gestation and then during postpartum hospitalization	n = 762 African-American	Prenatal care entry 4-6 months – Overall DRI: (crude OR 1.08; CI 0.95-1.23) and (adjusted OR 1.00; CI 0.87-1.14) – Denial of personal racism: (crude OR 1.05; CI 0.88-1.26) and (adjusted OR 0.95; CI 0.78-1.14) – Denial of group racism: (crude OR 1.24; CI 0.96-1.61) and (adjusted OR 1.12; CI 0.85-1.47) Prenatal care entry ≥ 7 months or no care – Overall DRI: (crude OR 1.20; CI 1.02-1.42) and (adjusted OR 1.19; CI 1.00-1.41) – Denial of personal racism: (crude OR 1.12; CI 0.89-1.42) and (adjusted OR 1.08; CI 0.84-1.38) – Denial of group racism: (crude OR 1.64; CI 1.20-2.25) and (adjusted OR 1.64; CI 1.18-2.28)
Bécares et al.,^29^ 2016/CS	Racial discrimination in relation to PN perceived stress	EODQ	Last trimester of pregnancy	n = 3,355 women	Personal attack: Physical attack ever: (coeff. 1.27; CI −0.18-2.72); physical attack past: (coeff. 1.14; CI −1.20-3.49) Verbal attack ever: (coeff. 1.68; CI 1.06-2.29); verbal attack past: (coeff. 1.62; CI 0.74-2.49) Any personal attack ever: (coeff. 1.68; CI 1.08-2.28); any personal attack past: (coeff. 1.50; CI 0.65-2.35) Unfair treatment: Healthcare professional ever: (coeff. 1.42; CI 0.44-2.39); healthcare professional past: (coeff. 1.79; CI 0.51-3.07) Work ever: (coeff. 2.08; CI 1.20-2.97); work past: (coeff. 1.23; CI −0.23-2.69) Housing ever: (coeff. 1.51; CI 0.58-2.44); housing past: (coeff. 2.27, CI 0.81-3.74) Criminal justice system ever: (coeff. 1.22; CI 0.05-2.38); criminal justice system past: (coeff. 1.25; CI −0.56-3.07) Banking system ever: (coeff. 2.55; CI 1.05-4.04); banking system past: (coeff. 1.21; CI −1.06-3.48) Educational system ever: (coeff. 0.98; CI 0.15-1.82); educational system past: (coeff. 1.75; CI −0.50-3.99) One experience ever: (coeff. 1.08, CI 0.43-1.73); one experience past: (coeff. 1.64; CI 0.84-2.44) Two or more experiences ever: (coeff. 2.65; CI 1.95-3.35); two or more experiences past: (coeff. 2.02; CI 0.91-3.12)
Slaughter-Acey et al.,^27^ 2019/CH	Racial macro-aggressions in relation to delayed PN care	20-item DLE-B	Interviewed 24-48 hours after delivery	-n = 909 first PN care -n = 300 no or late PN care	– African-American women with DLE-B score > 71 for no or late PNC: (unadjusted OR = 1.24; 95% CI = 0.95-1.61) and (adjusted OR = 1.31; 95% CI = 1.00-1.72) – Stratified according to maternal skin tone with DLE-B score > 71 for no or late PNC: light brown African-American women (adjusted OR = 1.67; 95% CI = 1.02- 2.71) and dark brown African-American women (adjusted OR = 2.29; 95% CI = 1.18-4.43)
Stancil et al.,^30^ 2000/CS	Racial discrimination in relation to pregnancy outcomes	EODQ	In first half and second half of pregnancy	n = 94 African-American	Racial discrimination - ever: 54.3% (51); applying for housing: 23.4% (22); applying for a job: 28.7% (27); at school: 26.6% (25); getting medical care: 7.4% (7); dealing with police or in court: 11.7% (11); at work: 28.7% (27); other: 7.4% (7)
Christian et al.,^35^ 2012/CS	Racial discrimination in relation to Epstein-Barr virus (EBV) reactivation in pregnancy and postpartum	EODQ	1st, 2nd and 3rd trimesters and at 4-9 weeks postpartum	n = 56 -n = 38 African-American -n = 18 white	– High versus low discrimination: higher EBV virus capsid antigen immunoglobulin G (VCA IgG) antibody titers during the first (P = 0.03) and second trimesters of pregnancy (P = 0.04); 3rd trimester (P = 0.12) and at postpartum (P = 0.06) – White vs African American women Higher EBV VCA IgG antibody titers at all three trimesters and at postpartum [high discrimination: P values < 0.001; low discrimination: P = 0.01 (1st), 0.001 (2nd), 0.002 (3rd) and 0.001 (postpartum)
Hilmert et al.,^36^ 2014/CS	Racism in relation to blood pressure changes during pregnancy	EODQ	22 to 24 weeks of gestation	-n = 39 African-American women	Diastolic blood pressure (DBP) change analyses: Racism in relation to changes in DBP interactions was significant in the analyses involving childhood: indirect racism (β = - 0.36; Δ*R* ^2^ = 0.12; P < 0.01); and childhood personal racism (β= −0.30; Δ*R* ^2^ = 0.07; P < 0.05), both showing the same pattern of associations depicted. Systolic blood pressure (SBP) change analyses. Parallel analyses on changes in SBP did not reveal any statistically significant results (all P values > 0.05).
Peters et al.,^37^ 2014/CS	African-American women's trust in provider during pregnancy	Trust in Physician Scale RaLES-Brief	Once a month during weeks 4-28; every 2 weeks during weeks 28-36 and every week from week 36 until birth	n = 189 African American women	Trust was inversely associated with previous experience of racism, specifically in healthcare (r = −0.16; P = 0.03), as women who reported experiencing racism in healthcare had significantly lower trust scores than women who did not report such an experience (t (187) = 2.17; P = 0.03)
Yang et al.,^38^ 2014/CS	Racial segregation in relation to maternal smoking during pregnancy	Racial Segregation Index	Pregnancy	County-level n = 2556 (NHW) (59%) (NHB) (16%) (NHA) (4%) (H) (21%).	Racial segregation index: NHB: living in a county where blacks are more segregated from whites was associated with higher probability of maternal smoking during pregnancy NHA: Asian women seemed to benefit more from living in a county where Asians were segregated from whites than in a county where these two racial groups were integrated H: Living in a Hispanic-white segregated community could be beneficial for Hispanic mothers
Attanasio et al.,^39^ 2015/CS	Perceived discrimination in maternity care	– 7 questions: during prenatal care (communication) – 3 questions: during birth hospitalization (Perceived discrimination domain)	Before, during and after recent birth	n = 2,231 -n = 1,308 NHW -n = 368 NHB -n = 555 H	Race: – NHB: (adjusted OR 2.99; CI 1.56-5.74) – H: (adjusted OR 2.25; CI 1.32-3.81)	Maternal health: – Pregnancy hypertension: (adjusted OR 2.41; CI 1.38-4.22) – Diabetes (adjusted OR 3.25; CI 2.09-5.04) – Obese pre-pregnancy (adjusted OR 0.63; CI 0.35-1.13)
Borders et al.,^40^ 2015/CH	Hormonal and inflammatory measurements of chronic stress during pregnancy according to racial discrimination scale	EODQ	14 and 22 weeks of gestation	Total: n = 112 – 55 NHB – 57 NHW	Krieger Discrimination Scale P < 0.001 – blacks: 11.3 ± 1.7 – whites: 13.3 ± 0.9 Association of mean stress biomarkers with race NHB women had significantly higher mean C-reactive protein levels in the second trimester (12.7 ± 11.9 versus 7.4 ± 8.3; P < 0.01) and third trimester (12.2 ± 14.9 versus 6.9 ± 7.4; P = 0.04) relative to NHW women. NHB women also had significantly higher adrenocorticotropic hormone levels in the second trimester (21.6 ± 11.9 versus 16.5 ± 8.5; P = 0.01) and third trimester (6.4 ± 15.1 versus 3.9 ± 4.0; P = 0.03) relative to NHW women. No differences in Epstein-Barr virus or corticotropin-releasing hormone levels were detected between the two racial/ethnic groups
Grobman et al.,^31^ 2016/CH	Psychosocial states and traits during pregnancy	EODQ	21 weeks of gestation.	n = 7,690	Krieger Racism ≥ 3: (%) - NHW: 2.0% - NHB: 21.0% - H: 13.2% - A: 15.8% - other: 15.8%; P < 0.0001
Grobman et al.,^32^ 2018/CH	Associations of preterm birth, hypertensive disease of pregnancy and SGA birth with self-reported measurements of psychosocial stress	EODQ	16 and 21 weeks of gestation	n = 8,962	Hypertensive disease of pregnancy – NHB (OR 0.98; CI 0.81-1.20) – H (OR 0.71; CI 0.58-0.86) – Asian (OR 0.82; CI 0.56-1.20) – Other (OR 0.85; CI 0.63-1.14) – Krieger > 3 (OR 0.81; CI 0.62-1.06)
Mendez et al.,^33^ 2020/CH	Racism in relation to pregnancy and postpartum	– TMDS – GRMS	18-32 weeks of gestation and at delivery	n = 230 -n = 146 white -n = 57 black	Only descriptive: black participants indicated more racism than white participants, and white participants indicated more sexism than black participants
Chambers et al.,^34^ 2020/CS	Racial discrimination among pregnant and postpartum black women	ICE (concentrations at the extremes) race + income measurement (formula)	Currently pregnant or early postpartum (6 weeks) with a singleton birth	n = 42 -n = 20 least deprived -n = 22 most deprived	Racial Discrimination – 93% of the women: at least one situational domain – 59.5% of the women: in three or more situational domains – The three most common situational domains were at school (59.5%), on the street or in a public setting (59.5%) and getting service in a store or restaurant (54.8%)
Francis et al.,^41^ 2017/CS	Racial discrimination in relation to perinatal sleep quality	EODQ	Each trimester and postpartum	n = 640 – PN n = 247 – Postnatal n = 393	Cross-sectional unadjusted analysis associations between discrimination and overall sleep quality: – Overall (overall sleep quality 0.058) - prenatal (overall sleep quality 0.042) - postpartum (overall sleep quality 0.076) – Black (overall sleep quality 0.048) – White (overall sleep quality 0.072)
Attanasio et al.,^18^ 2017/CS	Perceived discrimination during hospitalization for childbirth	Survey	8 weeks after birth	n = 2,400	Perceived discrimination during hospitalization for childbirth and non-attendance of postpartum visit (multivariate models) – Treated poorly due to race: (unadjusted OR 2.11; CI 1.25-3.57) and (adjusted OR 2.11; CI 1.15-3.87)

CS = cross-sectional study; CH = cohort study; PN = prenatal; EODQ = Experience of Discrimination Questionnaire; DRI = Denial of Racism Index; RaLES = Racism and Life Experiences Scale; DLE-B = Daily Life Experiences of Racism and Bother score; TMDS = The Major Discrimination Scale; GRMS = Gendered Racial Microaggressions Scale; NHB = non-Hispanic black; NHW = non-Hispanic white; H = Hispanic; OR = odds ratio; CI = confidence interval; coeff = coefficient; PNC = prenatal care; EBV = Epstein-Barr virus.

Fourteen studies assessed racial discrimination in relation to neonatal outcomes (Table 2) involving preterm birth (gestational age below 37 weeks) and low birthweight (less than 2500 grams).^32,36,42–53^

**Table 2:** Racism or racial discrimination assessed within studies on low birthweight and preterm infants

Author, year /study design	Subject	Measurement	Time of Interview	Sample size	Control group	Main results (descriptive or after crude/adjusted analysis)
Rosenberg et al.,^42^ 2002/CS	Racial discrimination in relation to premature birth	EODQ	Singleton births that had occurred in the previous two years.	n = 4,966 -n = 422 mothers of preterm babies -n = 4,544 mothers of full term babies	N/A	Preterm versus full term: – Job: (unadjusted OR 1.3; CI 1.1-1.7; adjusted OR 1.3; CI 1.1-1.6) – Housing: (unadjusted OR 1.0; CI 0.8-1.3; adjusted OR 1.0, CI 0.8-1.3) – Police: (unadjusted OR 1.2; CI 0.9-1.5; adjusted OR 1.1; CI 0.9-1.4) – Poorer service: (unadjusted OR 1.1; CI 0.7-1.5; adjusted OR 1.1; CI 0.7-1.5) – Not intelligent: (unadjusted OR 1.1; CI 0.9-1.5; adjusted OR 1.1; CI 0.8-1.4) – Causing fear: (unadjusted OR 1.4; CI 1.1-2.0; adjusted OR 1.4; CI 1.0-1.9) – Dishonest: (unadjusted OR 1.2; CI 0.8-1.7; adjusted OR 1.2; CI 0.8-1.7) – Worse than others: (unadjusted OR 1.2; CI 0.9-1.5; adjusted OR 1.1; CI 0.9-1.4) – Thinking about their race: (unadjusted OR 1.0; CI 0.7-1.4; adjusted OR 1.0; CI 0.7-1.4)
Mustillo et al.,^43^ 2004/CS	Self-reported racial discrimination in relation to differences in black and white preterm and low-birthweight deliveries	EODQ	Year 7 examination (1992–1993)	n = 352 – Black women n = 152 – White women n = 200	N/A	Preterm deliveries (n = 328) – Race/ethnicity: black versus white (OR 2.54; CI 1.33- 4.85) – Self-reported racial discrimination in 1 or 2: (OR 1.97; CI 0.89-4.38) – Self-reported racial discrimination in ≥ 3: (OR 2.42; CI 1.03-5.69).	Low birthweight deliveries (LR) (n = 320) – Race/ethnicity: black versus white (OR 4.24; CI 1.31-13.67) – Self-reported racial discrimination in 1 or 2: (OR 2.04; CI 0.50-8.31) – Self-reported racial discrimination in ≥ 3: (OR 4.81; CI 1.50-15.40)
Misra et al.,^44^ 2010/CH	Racism in relation to risk of preterm birth	RALES and RRS	22-28 weeks of gestation and postpartum	n = 843 African-American women	N/A	Racism and stress Lower stress: (unadjusted HR 0.88; CI 0.59-1.32) and (adjusted HR 0.92; CI 0.61-1.38). Higher stress: (unadjusted HR 1.29; CI 0.83-2.01) and (adjusted HR 1.30; CI 0.83-2.04).
Braveman et al.,^45^ 2017/CS	Racial discrimination in relation to preterm birth	Racial discrimination Chronic worry	4 months postpartum	Black women: n = 2,201 White women: n = 8,122	N/A	Chronic worry and racial discrimination unadjusted: – Black women: (PR 1.73; CI 1.12-2.67); - white women: (PR 1.77; CI 0.83-3.77)	Chronic worry, racial discrimination and social/demographic covariates: – Black women: (PR 1.95; CI 1.27-2.97); - white women: (PR 1.67; CI 0.73-3.79) Chronic worry, racial discrimination and social/demographic, behavioral and medical covariates: – Black women: (PR 2.00; CI 1.33-3.01); - white women: (PR 1.84; CI 0.91-3.71)
Bower et al.,^46^ 2018/CS	Racism in relation to preterm birth	ERQ	2-6 months postpartum	– n = 426 primiparous – n = 912 multiparous term birth – n = 268 multiparous preterm birth	N/A	Weighted population (crude) – Racism (OR 1.27; CI 1.04-1.54)	Adjusted for maternal age and BMI – Racism (OR 1.29; CI 1.04-1.59)
Fryer et al.,^47^ 2020/CS	Everyday discrimination in relation to preterm birth among African-American and Latina women	WSED	One month postpartum	n = 1,732 – 1,154 African-American – 578 Latina	N/A	Spontaneous preterm delivery – African-American (high discrimination): (unadjusted HR 1.4; CI 0.7-2.7) and (adjusted HR 1.5; CI 0.7-3.1) – Latina (high discrimination): (unadjusted HR 3.8; CI 0.9-15.1) and (adjusted HR 3.6; CI 0.9-14.4)
Wheeler et al.,^48^ 2018/CH	Racism in relation to preterm birth	PSS	Current pregnancy	n = 1,606 – n = 1,256 NHB – n = 350 NHW	N/A	Perceived racism score in relation to spontaneous preterm birth – Primiparous (adjusted OR 1.29; CI 0.91-1.83) – Multiparous term birth (adjusted OR 1.01; CI 0.79-1.30) – Multiparous preterm birth (adjusted OR 1.05; CI 0.78-1.40)	Perceived racism score in relation to spontaneous preterm birth among black women – Primiparous (adjusted OR 1.36; CI 0.93-1.96) – Multiparous term birth (adjusted OR 1.03; CI 0.80-1.31) – Multiparous preterm birth (adjusted OR 0.98; CI 0.72-1.35)
Grobman et al.,^32^ 2018/CH	Associations of preterm birth and SGA birth with self-reported measurements of psychosocial stress	ERQ	16-21 weeks of gestation	n = 8,962 women	N/A	Any preterm birth -NHB (OR 1.31; CI 1.04-1.64); - H (OR 0.95; CI 0.76-1.20); - Asian (OR 0.87; CI 0.56-1.36); - other (OR 1.14; CI 0.82-1.59); - Krieger > 3 (OR 0.91; CI 0.67-1.23)	Small for gestational age -NHB (OR 2.07; CI 1.69-2.53); -H (OR 1.45; CI 1.19-1.77); - Asian (OR 2.08; CI 1.54-2.81); - other (OR 1.42; CI 1.05-1.93); - Krieger > 3 (OR 1.01; CI 0.78-1.31)
Slaughter-Acey et al.,^49^ 2016/CH	Racism in relation to risk of preterm birth	DLE-B	24 and 48 hours after delivery	n = 1,232 African-American women	N/A	Mild to moderate depressive symptoms: perceived racism was significantly associated with preterm birth Severe depressive symptoms: perceived racism was not associated with preterm birth
Brown et al.,^50^ 2019/CH	Perceived discrimination in birth outcomes among women giving birth to an Aboriginal baby	4-item questions adapted from MIRE	When women's infant was 4-12 months old.	n = 344 women	N/A	Discrimination report – < 37 weeks: (unadjusted OR 1.0; CI 0.5-1.9) and (adjusted OR 1.1; CI 0.5-2.1) – SGA: (unadjusted OR 2.0; CI 1.1-3.6) and (adjusted OR 1.7; CI 0.9-3.2)	Discrimination report – < 2500 grams: (unadjusted OR 2.1; CI 1.1-4.1) and (adjusted OR 2.0; CI 1.0-3.9)
Lespinasse et al.,^51^ 2004/CC	Racial discrimination in relation to birthweight of infants of African-American women	ERQ	3 days after delivery.	-n = 104 mothers (low birthweight)	- n = 208 mothers (normal birthweight)	Exposure to racial discrimination (%OR): – 1 or more domain: (OR 1.9; CI 1.2-3.0) – 3 or more domains: (OR 2.7; CI 1.3-5.4)
Collins et al.,^52^ 2004/CC	Maternal exposure to interpersonal racial discrimination in relation to infants with very low birthweight	ERQ	72 hours after infants’ admission to the neonatal intensive care unit or nursery	-n = 104 African American infants (< 1500 g), born preterm (< 37 weeks)	-n = 208 African American women with term infants	Reported racial discrimination incidents Lifetime: – Job: (OR 3.0; CI 1.6-5.4); - at work: (OR 2.0; CI 1.1-3.5); - at school: (OR 1.9; CI 1.0-3.7); - public settings: (OR 1.4; CI 0.8-2.3); - medical care: (OR 0.9; CI 0.3-2.7) – ≥ 1: (OR 1.9; CI 1.2-3.1); - ≥ 2: (OR 2.1; CI 1.2-3.8); - ≥3: (OR 3.2; CI 1.5-6.6) – Adjusted ≥ 1 domain: OR 1.7; CI 1.0-9.2 – Adjusted ≥ 3 domains: OR 2.6; CI 1.2- 5.3
Dominguez et al.,^53^ 2008/CH	Racism as predictor of birthweight	ERQ	24-26 weeks of gestation	-n = 51 African American women -n = 73 NHW	N/A	Race step 1 (β = −0.25; P < 0.05), with African American infants weighing an average of 280.84 g less than white infants. Perceived racism lifetime score step 2: each unit increase in lifetime perceived racism was associated with a 39.59-g decrease in birth weight. Interaction term (step 3) -Childhood-direct racism (β = 0.17; P < 0.10). Each unit increase was associated with a 137.10-g increase in birth weight -Childhood-vicarious racism (β = - 0.25; P < 0.01). Each unit increase was associated with a 167.85-g decrease in birth weight.
Hilmert et al.,^36^ 2014/CS	Association between birthweight and racism	ERQ	22 to 24 weeks of gestation	n = 39 African American pregnant women	N/A	Childhood indirect, adulthood personal and total racism exposure: significant amount of variance in birth weight (all P values < 0.05). Association between adjusted birthweight and childhood indirect racism (β = −0.24) was not significant (P > 0.10). Association between birthweight and total racism (β = −0.27) was slightly significant (P < 0.10).

CS = cross-sectional study; CH = cohort study; CC = case-control study; EODQ = Experience of Discrimination Questionnaire; SGA = Small for Gestational Age; MIRE = Measure of Indigenous Racism Experience; RRS = Racism-Related Scale; WSED = Williams Scale of Everyday Discrimination; RaLES = Racism and Life Experiences Scale; DLE-B = Daily Life Experiences of Racism and Bother score; PSS = Perceived Stress Scale; ERQ = Experiences of Racism Questionnaire; NHB = non-Hispanic black; NHW = non-Hispanic white; H = Hispanic; OR = odds ratio; CI = confidence interval; HR = hazard ratio; PR = prevalence ratio; N/A = not applied.

**Figure 2:**
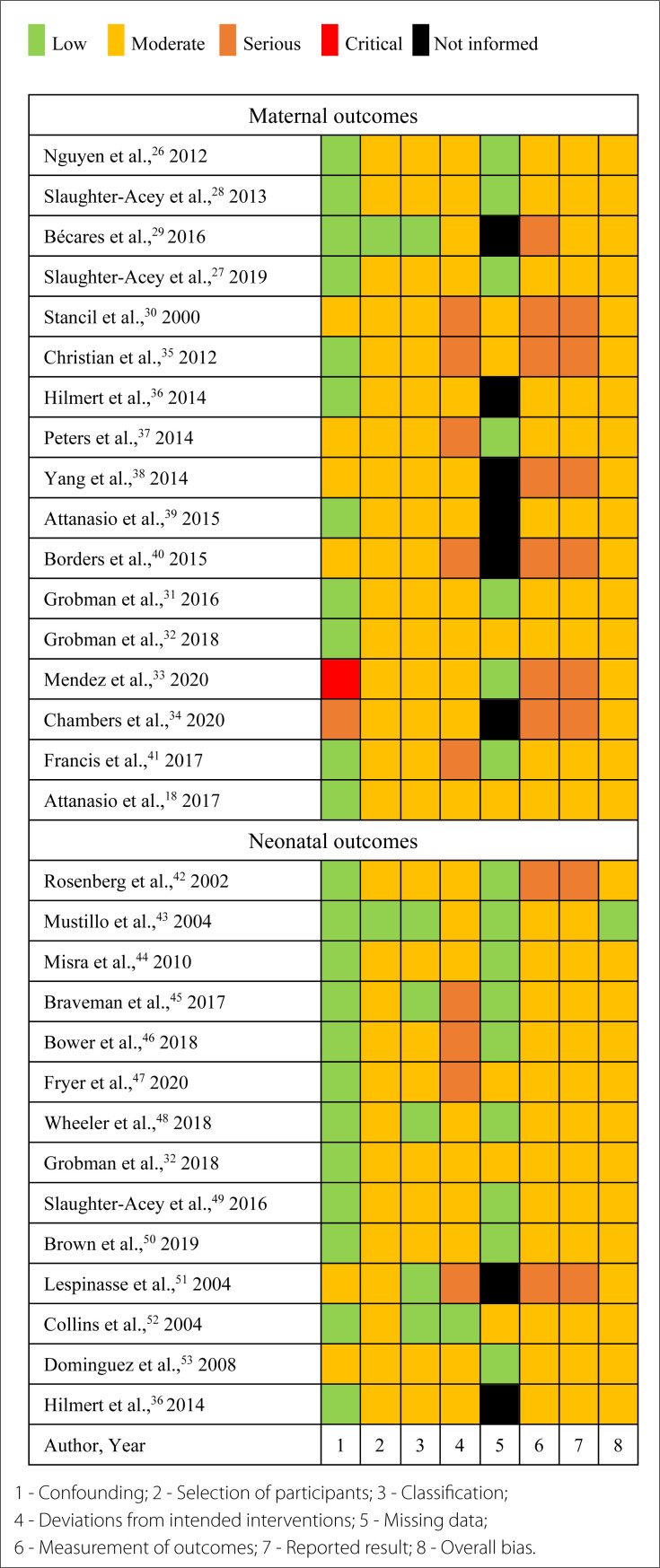
ROBINS-I tool (Risk Of Bias In Non-randomized Studies – of Interventions) applied to the studies included.

The risk of bias of the studies included is described in Figure 2.^18,28–53^ The overall classification of bias in these studies was moderate. Overall bias was classified as a moderate risk of bias in all 29 studies.

### Pregnancy

Two studies assessed the association between racial discrimination and maternal smoking. Nguyen et al.^26^ described experiences of discrimination as a predictor for smoking during pregnancy. They found that women who experienced high levels of discrimination (≥ 3 domains) were 2.6 times (odds ratio, OR 2.64; confidence interval, CI 1.25 to 5.60) more likely to smoke during pregnancy. When stratified according to race, black women reporting high levels of discrimination were 3.4 times (OR 3.36; CI 1.23 to 9.19) more likely to smoke during pregnancy than Hispanic women. Yang et al.^38^ reported a higher probability of maternal smoking during pregnancy when black women were less integrated into society at large than non-Hispanic whites were.

Slaughter-Acey et al.^28^ investigated the indices of denial of racism in antenatal care for African-American women; they found that the overall denial of racism index was 19% higher (adjusted odds ratio, AOR 1.19; CI 1.00–1.41) for African-American women with no prenatal care or late to antenatal care (attendance at ≥ seven months of gestation), compared with early prenatal care attendance (attendance at ≤ three months of gestation).

Becares et al.^29^ reported on lifetime and past-year experiences of racial discrimination covering personal attacks and unfair treatment in a group of multiple-ethnicity women categorized as Māori, Pacific, Asian and European. Lifetime and past-year experiences of racial discrimination with any unfair treatment were more common among Māori women; however, they were highly prevalent among all non-European mothers.

Slaughter-Acey et al.^27^ revealed that African-American women with Daily Life Experience of Racism and Bother score > 71 were 31% more likely to present delayed antenatal care than non-African-American women. Fifty-one (54.3%) out of 94 African-American women reported experiences of racial discrimination in a study by Stancil et al.^30^ Of these, 28.7% reported these experiences while applying for a job and 28.7% reported that these were occurrences at work.

Christian et al.^35^ investigated the association between racial discrimination and Epstein-Barr virus capsid antigen immunoglobulin G during pregnancy and postpartum. Epstein-Barr virus capsid antigen immunoglobulin G antibody titers were significantly higher during the first (P = 0.03) and second trimesters of pregnancy (P = 0.04) in women reporting high levels of racial discrimination, compared with those reporting low racial discrimination.

Two studies by Grobman et al.^31,32^ were selected. One study showed that non-Hispanic black women were more likely to perceive racism and with the least social support. In the other, no association was observed between race-ethnicity and hypertensive disease of pregnancy.^36^ Mendez et al.^33^ used smartphone technology to assess exposure to racism and found that black women experienced more racism than white women. Peters et al.^37^ investigated African-American women's trust in providers during pregnancy. Trust was negatively correlated with previous experience of racism (r = −0.16; P = 0.03).

In the study by Attanasio et al.,^39^ black and Hispanic race/ethnicity were found to be associated, respectively, with threefold and twofold increases in perceived racial discrimination. Borders et al.^40^ found an association between stress biomarkers and race. Non-Hispanic black women presented significantly higher adrenocorticotropic hormone and C-reactive protein levels in the second and third trimesters, in comparison with non-Hispanic whites.

Chambers et al.^34^ described racial discrimination in nine situations. 93% of the women reported racial discrimination in at least one situational domain and the three most frequent ones were at school (59.5%), on the street or in a public setting (59.5%) and getting service in a store or restaurant (54.8%). Lastly, perinatal sleep quality was studied and correlated with racial discrimination in the study by Francis et al.^41^ This positive association showed that greater reported everyday racial discrimination was associated with poorer overall sleep quality.

### Childbirth and postpartum period

Attanasio et al.^18^ investigated perceived discrimination in relation to hospitalization for childbirth and non-attendance of post-partum visit. Women who reported racial discrimination were more than twice as likely to miss their postpartum visit, compared with women who did not report this type of discrimination (AOR 2.11; CI 1.15–3.87).

### Preterm birth

Rosenberg et al.^42^ showed that preterm birth occurred 30% more often among women who reported unfair treatment on the job and 40% more often among women who reported that people acted fearfully in relation to them at least once a week.

Mustillo et al.^43^ showed that black women were 2.5 times more likely to have a preterm birth than white women. Women who reported having three or more experiences of racial discrimination were 2.4 times more likely to have a preterm birth than those who did not report racial discrimination. Similarly, Braveman et al.^45^ reported that racial discrimination was significantly associated with preterm birth among black women before (prevalence ratio, PR 1.73; CI 1.12–2.67) and after (PR 2.00; CI 1.33–3.01) adjustment for social/demographic, behavioral and medical covariates. Preterm birth was also associated with experiences of racism, with a 29% increased risk.^46^

Grobman et al.^32^ found that non-Hispanic black women experiencing racism were at higher risk of any preterm birth and of small-for-gestational-age birth, compared with non-Hispanic white women. Similarly, Hispanic and Asian women experiencing racism were also at risk of small-for-gestational-age birth.

In four studies, exposure to racial discrimination did not interfere in the frequency of preterm birth among black women.^44,47–49^ On the other hand, Fryer et al.^47^ showed that Latina women presented a significant association between racial discrimination and preterm birth. Moreover, in the study by Brown et al.,^50^ Aboriginal women who experienced racial discrimination in perinatal care showed a 90% higher risk of having an infant who was small for gestational age. They did not find any association with preterm birth in their sample.

### Birthweight

Two case-control studies found an association between very low birthweight and maternal exposure to racial discrimination. Very low birthweight was associated with incidents of lifetime exposure to interpersonal racism in three or more domains of the racial discrimination questionnaire (AOR 2.6; CI 1.2–5.3).^52^ Exposure to racial discrimination perceived in three or more domains of the racial discrimination questionnaire and being alone in the delivery room were associated with a twofold greater chance of having an infant with very low birthweight (OR 2.7; CI 1.3–5.4).^51^

Mustillo et al.^43^ found a strong association between racial discrimination and birthweight. Black women were over four times more likely to deliver low birthweight infants than white women. Moreover, women reporting elevated levels of racial discrimination were almost five times more likely to deliver a low birthweight infant than women who did not report racial discrimination.

Dominguez et al.^53^ reported that each unit increase in the perception of racial discrimination over women's lifetimes was associated with a 39.59-gram decrease in infant birthweight. Furthermore, childhood-vicarious racism (i.e. indirect exposure to prejudice and discrimination) was a significant predictor of decreased birthweight.

Hilmert et al.^36^ also analyzed the involvement of racism in birth-weight. In their interview method, adapted from Krieger et al.,^12^ they included subscales for direct and indirect exposure during childhood (≤ 16 years) and in adulthood (> 16 years). Correlation analyses showed that childhood indirect, adulthood personal and total racism exposure demonstrated significant amounts of variance in birth weight (all P-values < 0.05). After including control variables, the association between adjusted birthweight and indirect racism during childhood (β = −0.24) ceased to be significant (P> 0.10).

Lastly, Brown et al.^50^ revealed that women who experienced racial discrimination in perinatal care were 90% more likely to have a baby with low birthweight than were women who did not experience such discrimination.

## DISCUSSION

This review found that perceived racism or racial discrimination was negatively associated with maternal and neonatal outcomes. It supports the reality that racism is a public health problem that warrants significant discussion with the goal of finding practical solutions through implementation of anti-racist measures.

This review also demonstrated that women experiencing racial discrimination were more likely to present poorer maternal health outcomes during pregnancy and childbirth and in the postpartum period. Trust in providers was compromised during pregnancy; it was inversely associated with previous experiences of racism. Racial discrimination during antenatal care was associated with later onset of antenatal visits or lack of attendance of postpartum visits. It was also associated with smoking, which is a well-known risk factor for poor health outcomes.^54^ Stress biomarkers also presented elevated during the second and third trimester among African-American women. Epstein-Barr virus immunoglobulin G (IgG) antibody titers were significantly elevated in women reporting high levels of racial discrimination. African-American women were found to have elevated antibody titers throughout pregnancy and the postpartum period. There is research supporting the notion that maternal stress before and during pregnancy is associated with poor pregnancy outcomes, including low birthweight, preterm birth and infant mortality.^55^

Racial discrimination also plays a negative role in pregnancy blood pressure. Pre-pregnancy hypertension and diabetes were associated with higher odds of perceived racial discrimination. Childhood exposure to racism presented a significant association with change in diastolic blood pressure in African-American women. High blood pressure during pregnancy is associated with pregnancy complications, such as preeclampsia, cesarean delivery, preterm delivery, low birth weight, neonatal intensive care admission and perinatal death.^56^

Racism appears to be a risk factor for worse neonatal outcomes, with greater occurrence of low birthweight and preterm birth. Racial discrimination was also significantly associated with premature birth in most, but not all the studies on this subject.

One major strength of our study was that it used a defined search strategy and predetermined eligibility criteria. We included studies that measured racial discrimination using an instrument that showed some association with obstetric outcomes, unlike previous studies, in which disparities or inequities between groups of women were reported but no mention of the racism or racial discrimination suffered by these women was made. We highlighted the social determinants of maternal and neonatal health: specifically, exposure to stress or stressors and social relationships and interactions that influence health outcomes, such as racism or racial discrimination.^57–60^

On the other hand, this review presented several limitations. In addition to methodological problems, the interventions and outcomes differentiated substantially among the studies included. Comparison among those studies would induce bias and the results would need to be interpreted with caution. The use of thirteen different questionnaires limited the possibility of performing a meta-analysis. The existence of thirteen different questionnaires also points to the need for further study on this topic and definition of the best instruments for its evaluation. The limitations on the use of scales for questions that assess personal experience are widely known. However, even with these limitations, use of scales provides the means to take the first step towards knowledge of issues that are more personal and cultural.

## CONCLUSION

Perceived racism presented an association with poor obstetric outcomes. In summary, even with the stated limitations to these studies, a prompt response from society is urged, in order to be attentive to prevention of racism in all healthcare spaces. Our institution, peers, trainees and patients need to engage in anti-racist training. Anti-racist measures are needed so as to address the problems that are causing patients to perceive or experience racism. These measures should ultimately contribute to reduction of racial disparities in obstetric outcomes.
